# Integrated analysis of dysregulated lncRNA expression in breast cancer cell identified by RNA-seq study

**DOI:** 10.1016/j.ncrna.2016.09.002

**Published:** 2016-09-21

**Authors:** Rashmi Tripathi, Apoorva Soni, Pritish Kumar Varadwaj

**Affiliations:** a Department of Bioinformatics, Indian Institute of Information Technology, Allahabad, U.P, India; b Department of Molecular and Cellular Engineering, Sam Higginbottom Institute of Agriculture, Technology and Sciences, Allahabad, U.P, India

**Keywords:** RNA sequencing, Transcriptomics, Differentially expressed genes, Long non-coding RNAs (lncRNAs), Breast cancer, Dysregulation

## Abstract

Among all the sequencing techniques, RNA sequencing (RNA-seq) has galloped with pace adopting the profiling of transcriptomic data in almost every biological analytics area like gene regulation study, development biology and clinical research. Recently the discovery of differentially expressed genes across different conditions has outshone the barrier of genetic & epigenetic regulations. The present work identified and analyzed differentially expressed novel long non-coding RNAs (lncRNAs) for breast cancer. A complex computational pipeline was adopted for the study which includes analysis of 18498 differentially expressed genes with 4114 up-regulated and 3475 down-regulated transcripts. The overexpression of lnc-MTAP (*CDKN2B-AS1*), lnc-PCP4 (*DSCAM-S1*), and lnc-FAM (*H19)* in breast cells suggests that these lncRNAs may have significant role to play in breast cancer. These results validated the relevance of the dysregulation pattern in cancer cells due to the presence of lncRNAs. The study further opens a new scope for experimental analysis to confirm the aberrant expression pattern of these lncRNAs which may act as potential bio-markers for the diagnosis and early detection of breast cancer.

## 1 Introduction

**Fig. 1:**
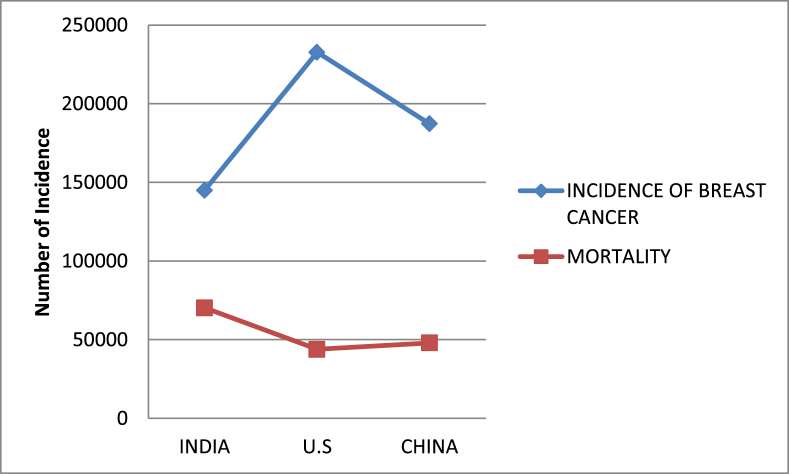
Statistics for breast cancer in India, U.S. and China for the Year 2012 (Adopted from WHO, International Agency for Research on Cancer).

Breast Cancer is second leading cause of morbidity and mortality worldwide, prevalently affecting female population [1]. According to the *World Cancer Research Fund International* 2012 report, U.S, China and India shares almost one third burden of the disease (accounting for approximately 25% of all new cancer) cases diagnosed [2]. Globally 0.45 million patients die from breast cancer annually, which solely constitutes 13.7% of female cancer deaths [3]. Breaking the myth that the disease widely flourishes in the developed countries, 144937 incidences of breast cancer and 70218 cases of death due to breast cancer was reported in Indian continent itself, as accounted by the latest survey done by *Globocan 2012*. The disease has outnumbered the cases of cervical cancer among women in India [4]. The statistics report shown in Fig. 1 depicts the incidence of breast cancer and mortality rate in three countries, viz. India, U.S and China.

Breast cancer can be divided into different types, the most common being the carcinoma (also summoned as adenocarcinoma). Other type of cancer occurring in the breast is sarcoma [5]. Depending upon the invasiveness and non-invasiveness and targeting areas (lobular and ductal), carcinoma can be characterized as invasive (or infiltrating) ductal carcinoma (IDC), ductal carcinoma *in situ* (DCIS), and invasive lobular carcinoma (ILC) [6], [7]. Sometimes tumour can be the result of mixture of different types such as invasive cancer and *in situ* cancer occurring in single breast tumour. In 2014, 232670 new cases of invasive breast cancer and 62570 new cases of *in situ* breast cancer (which includes DCIS and ILC) were reported by American Cancer Society. Statistics show that breast cancer has devastating effect on our health and represents about 90% of all the oral malignancies [8]. Early detection of cancer is crucial as treatment of late stage cancer is often difficult in less developed settings. Therefore, recent technologies like high-throughput sequencing can act as strong play card in the run [9].

The dawn of Next Generation Sequencing (NGS) has miraculously revolutionised the study of cancer genomics and molecular biology. Moreover, NGS has appeared as an effective way to capture large amount of genomic information related to diseases [10] by statistical study of clinical workflow. The study can produce snapshot of the actively expressed genes and the transcripts that has metamorphose the expression analysis experiments by providing in-depth identification of gene isoforms, splice junctions, unique transcripts and translocation events, specific alleles and post transcriptional base modifications in a cost effective way with unprecedented sequencing speed and accuracy [11]. Among all the sequencing techniques, RNA sequencing (RNA-seq) has galloped with pace adopting the profiling of transcriptomic data in almost all the biological specified areas like gene regulation study, development biology and clinical research [12], [13]. The discovery of differentially expressed genes (DEGs) in diverse contrasting conditions (such as, stimulated versus unstimulated or wild type versus mutant or knockdown versus control or normal versus tumour) has helped in the identification of underlying mechanism of the disease, out showing the barrier of genetic & epigenetic regulations [14].

Functionally, the annotation of the transcriptional activity including enumerable coding and non-coding genes profiling at once is important to construct an overall image of the cell activity [15]. The selection of such DEGs is on the basis of the amalgamation of score cut-off and expression change threshold that depends on *P* values obtained by statistical modelling.

Millions of short sequences (reads) are generated by RNA-sequencing technique. These reads are aligned to a reference genome and the number of reads aligning within a genomic feature of interest is used to measure the enrichment of the characteristic in the dataset [16], [17].

A class of non-coding RNAs (ncRNAs) that does not encode for protein includes, *tRNA* (transfer RNA)- which is involved in protein synthesis by actively participating in decoding of nucleic acid language and protein language, and *rRNA* (ribosomal RNA)- which work as protein synthesis factories in the cell. The complex association of tRNA and mRNA like regions forms another class of ncNA known as *tmRNA*. The tmRNA's quality control system monitors protein synthesis and releases ribosomes stalled during translation and target the nascent polypeptides for degradation. Other class of ncRNAs include *snRNA* (small nuclear RNA), found in the nucleus of eukaryotic cell, involved in RNA splicing and maintaining the telomers, and *snoRNA* (small nucleolar RNA) which play important role in chemical modifications such as RNA methylation, and formation of small nucleolar ribonucleoprotein (snoRNP) [18]. A group of small silencing RNAs which are molecules of approximately 20 nucleotides in length, forms union with Argonaute protein family members includes *miRNA* (micro RNA), *siRNA* (small interfering RNA) and *piRNA* (Piwi-interacting RNA). In recent years, the attention from short ncRNAs has shifted towards long ncRNAs (*lncRNAs*). It has become evident that mammalian genomes principally encode lncRNAs, the group which is rapidly gaining prominence [19], [20].

**Fig. 2:**
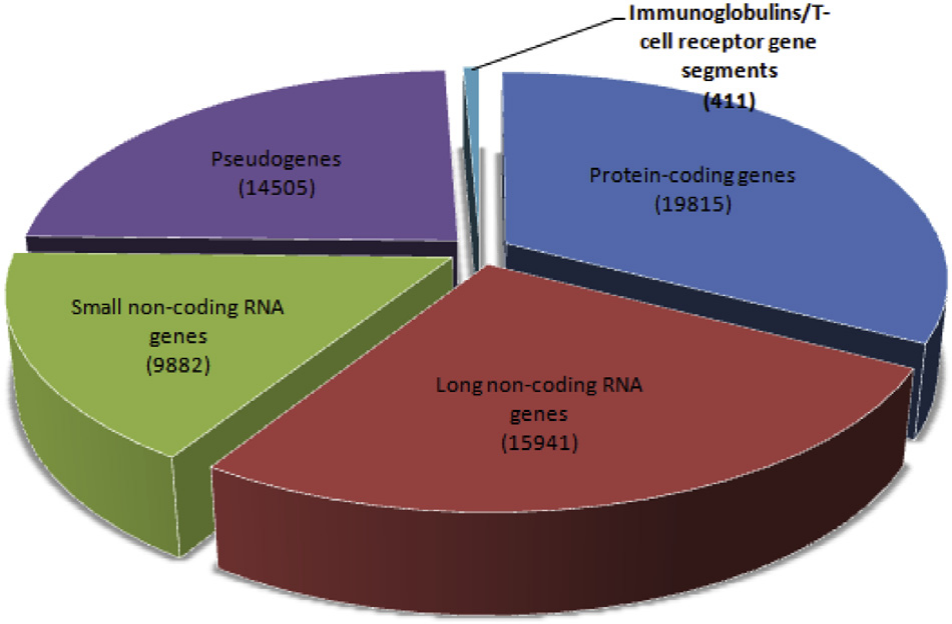
Statistics about the current Human GENCODE Release (version 24: August 2015 freeze, GRCh38) states that there are total 60,554 genes. The major category is of 25,823 non-coding RNAs genes (both, small non-coding RNAs and long non-coding RNAs).

**Fig. 3:**
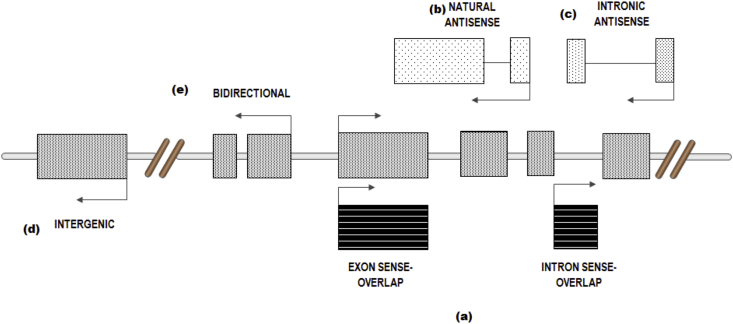
Schematic diagram illustrating the origin of long non-coding transcripts based on their biogenesis. lncRNAs can be classified on the basis of their proximity to protein coding genes (PCGs) as (a) *exon sense* & *intron sense* (which overlaps the sense strand of PCG), (b) *natural antisense* (which overlaps the antisense strand of PCG), (c) *intronic antisense* (which is derived entirely from or within an intron of another transcript), (d) *intergenic* (which is not located near any other protein coding loci), and (e) *bidirectional* (which have transcription start sites in close proximity to a PCG but are transcribed in the opposite direction) [21].

Once tagged as “junk”, the lncRNA clearly demonstrates that they can perform different functions more than being a messenger. Clear insights can end up the research at the “*RNA-level*” extracting information from the “*hidden*-*jewel*” ignoring the movement towards the next-protein level. LncRNAs are endogenous cellular RNAs which lack significant positive strand open reading frame (ORF), *i.e.* lack protein coding potential. These are of more than 200 nucleotides in length and are distinct from any known functional RNA classes [21]. This group of ncRNA does not constitute the homogeneous class of functionally related molecules [22]. The GENCODE 24 release catalog is much larger and expansive than previously expected accounting for approximately 15,941 lncRNAs in human genome as shown in Fig. 2 [23]. Fig. 3 shows five types of lncRNAs classified on the basis of their biogenesis.

While lncRNAs are pervasively transcribed in the genome, their potential involvement in human disease is not yet understood. Several lncRNAs can regulate gene expression at various levels, including chromatin modification, transcription, and post-transcriptional processing [24]. RNA-seq analysis can identify the lncRNAs related to different cancers. Dysregulation of lncRNAs is related to prognosis, metastasis, and recurrence in different cancer types affecting several processes related to oncogenesis, including cell growth and proliferation [25]. The over-expression of some lncRNAs (such as *HOTAIR, MALAT1, BCYRN1*, *etc*.) with proto-oncogenic function in normal cell increases tumour growth and matrix invasion of cancer cells. Some lncRNAs, such as the *XIST* (X inactive-specific transcript) or *HOTAIR*, forms heterochromatin structure and interact with chromatin modelling complexes actively altering the expression of their target genes [26], [27]. Other lncRNAs regulate the transcriptional activity by the process of RNA-binding proteins, acting as co-activator of TFs, or repressing a major promoter of their target genes [28].

Here we conducted a comprehensive study of lncRNA expression profiles across two cell lines- MCF10A and MCF7 using RNA-seq technique. We grouped our analysis into two categories *(1) pre-process-and-identify,* and *(2) annotate-and-analyse*, based on the ways we deal with the reads (small fragments of DNA) obtained from a sequencer. The first category does the pre-processing of the raw data, and then counts the number of reads that fall within condition specific boundaries. The second category seeks to identify the novel lncRNAs and related information about the relative expression pattern, followed by deep analysis of the non-coding materials.

## 2 Methods

### 2.1 NGS datasets and sample information

**Fig. 4:**
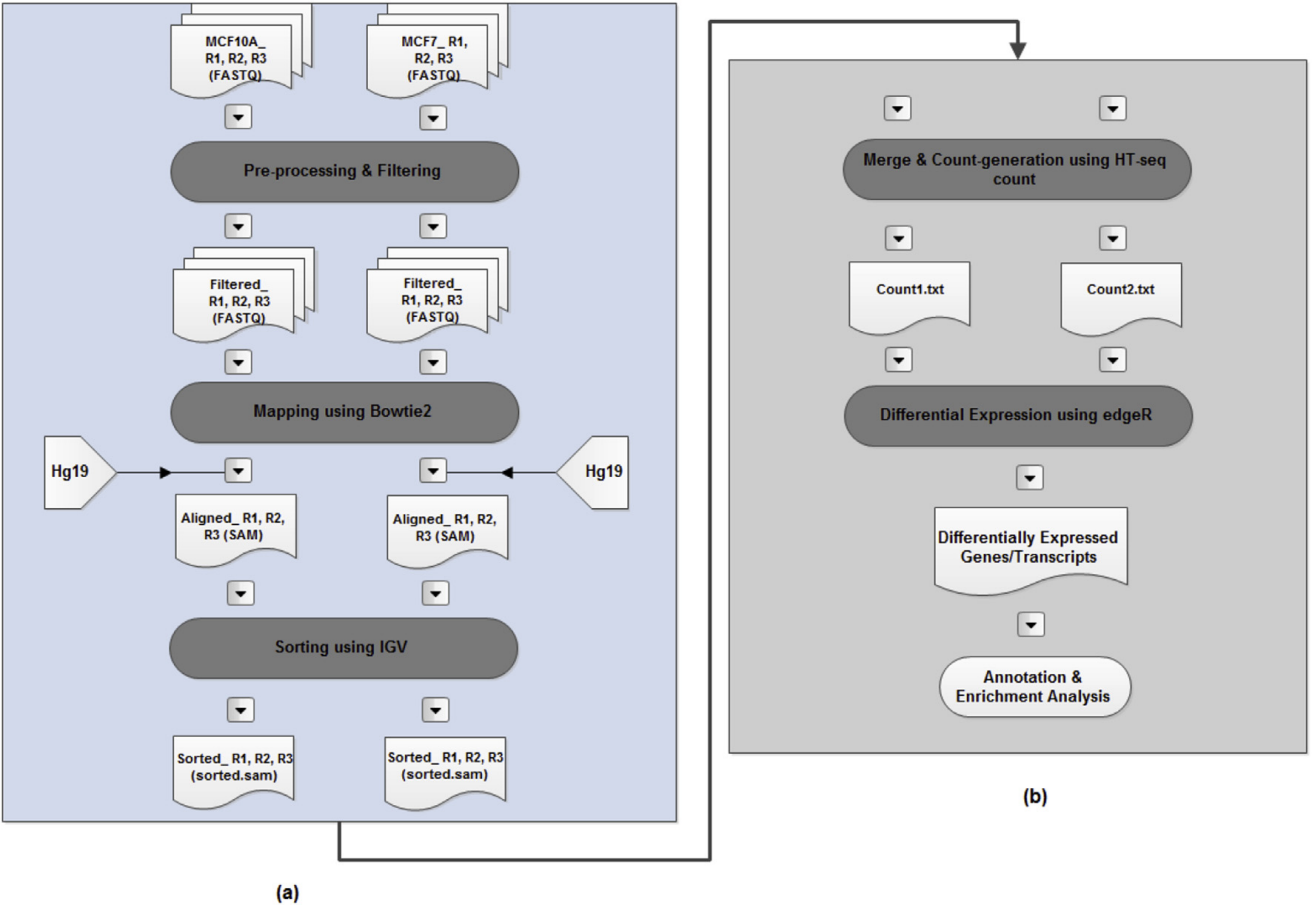
RNA-seq workflow. The input consists of sequencing reads (FASTQ files), a reference human genome sequence (FASTA file), and gene annotations (GTF and GFF files). The different stages of RNA-seq's workflow are illustrated and are grouped into two categories (a) *pre-process-and-identify,* and (b) *annotate-and-analyse*.

The comprehensive analysis was performed on two contrasting dataset (*i.e.* normal-like mammary epithelial cell line and transformed oestrogen responsive breast cancer cell line derived from a metastatic site) in order to characterize genome organisation during breast cancer development by repurposing the previously published gene expression profiles. The primary NGS datasets generated using Illumina's HiSeq 1500 platform and related RNA-seq library clinical information was obtained from Gene expression omnibus (GEO) database (http://www.ncbi.nlm.nih.gov/geo/query/acc.cgi?acc=GSE71862). The lncRNA NGS dataset comprised of SE (single-end) 36bp length sequence of 6 samples including 3 normal cells as control and 3 cancer cells as treated (Table 1). Both the data sets were analyzed using RNA-seq techniques complete workflow. Fig. 4 gives the overall steps involved in gene expression analysis. A protocol was developed, from filtering, mapping of reads, to count generation, to the discovery of DEGs, with a strenuous prominence on quality checks throughout the workflow. Entire computational analysis was carried out on CentOS based HP server with 48 cores 2.2 Ghz AMD processors configuration and 256 GB random access memory (RAM) as well as Ubuntu Linux.

**Table 1:** Clinical features of all 6 dataset (3 replicates of each normal cell line and treated cell line).

Replicates	GSM ID	Size (in SRA format)	Layout
Normal cell line			
MCF10A_RNA-Seq_R1	GSM1847015	1.1 Gb	Single end
MCF10A_RNA-Seq_R2	GSM1847016	1.1 Gb	Single end
MCF10A_RNA-Seq_R3	GSM1847017	1.9 Gb	Single end
Treated cell line			
MCF7_RNA-Seq_R1	GSM1847018	1 Gb	Single end
MCF7_RNA-Seq_R2	GSM1847019	1.9 Gb	Single end
MCF7_RNA-Seq_R3	GSM1847020	1.8 Gb	Single end

Computers with 8 cores 2.5 Ghz Intel processors and 8 GB RAM.

### 2.2 RNA-sequencing and lncRNA profile mining

The SE 36bp raw sequenced reads obtained from Illumina were stored in SRA format in the GEO database and was processed using RNA-seq workflow (http://www.ncbi.nlm.nih.gov/sra). The raw files embrace millions or billions of short sequence reads downloaded in SRA format and were directly converted and stored in FASTQ format using SRA toolkit (v2.3.2) (http://www.ncbi.nlm.nih.gov/Traces/sra/?view=software). The total read numbers in the library were 59941107 and 67020239 for 3 control and 3 treated conditions, respectively. Analysis required initial checks on sequence quality using read filtering tool FastQC (v0.11.5) (http://www.bioinformatics.babraham.ac.uk/projects/fastqc/) and filteR (Illumina1.8+ & Illumina1.3) (http://scbb.ihbt.res.in/SCBB_dept/Software.php) for removing noise. Filtering and trimming removed low quality reads (or bad reads) present due to genomic DNA contamination during the sample isolation and incompletely processed RNA (sequencer error) [29]. Filtering for quality and contamination check, removed total of 43.06% reads from control dataset (34.6 Gb of raw sequence) and 40.75% reads from treated dataset (37.3 Gb of raw sequence). Reads were mapped to the human reference genome (hg19) using splice-aware aligner Bowtie2 (v2.2.9) with default settings for subsequent analysis. For typical RNA-seq analysis (interested in identification of novel splice variants or new transcripts), using a splice-aware mapping is mostly preferred. After coarse filtration, best aligned short reads accounted for approximately 84.8% (control) and 90.33% (treated) using Bowtie2 (http://bowtie-bio.sourceforge.net/bowtie2/index.shtml) aligning exactly 1 and more than 1 times to the reference genome. From the set of mapped reads, sorted files in SAM format which were generated using IGV- Integrative Genomics Viewer (https://www.broadinstitute.org/igv/) tools, were merged and counted. The count numbers were later assembled into a table (features information were stored in rows and sample related information were present in columns) using HT-seq count (v0.5.4) (http://www-huber.embl.de/HTSeq/doc/install.html) (Table 2).

**Table 2:** Summary of transcriptomics data generated on Illumina Genome Analyzer IIx obtained through the process of RNA-sequencing.

	GSM1847015	GSM1847016	GSM1847017	GSM1847018	GSM1847019	GSM1847020
Total number of single end reads	17070793	15553889	27316425	14406303	27004244	25609692
Percentage of reads removed after quality filtering	25.17	21.58	53.23	21.7	41.44	36.84
Percentage of mapped reads (1 time)	56.25	53.07	51.44	47.9	61.01	50.78
Percentage of mapped reads (>1 time)	28.56	33.19	30.81	42.43	28.83	35.01

### 2.3 Statistical analysis for the identification and characterization of DEGs

For identifying DEGs, the statistical methods make the use of feature count table generated using HT-seq count tool [30]. Count feature based tools normalizes the log-summarized values using averaging algorithm for each gene set using formula shown in Eq. (1). The two condition specific libraries served as replicates, offering better confidence and higher coverage.

(1) $$RPKM=\frac{10^{9}\times \text{C}}{\text{N }\times \text{L}}$$

where, C is that number of reads which matches to a particular gene or gene segment, N is overall mapping reads in the experiment, and L is exon length of a gene (given in base pairs).

**Fig. 5:**
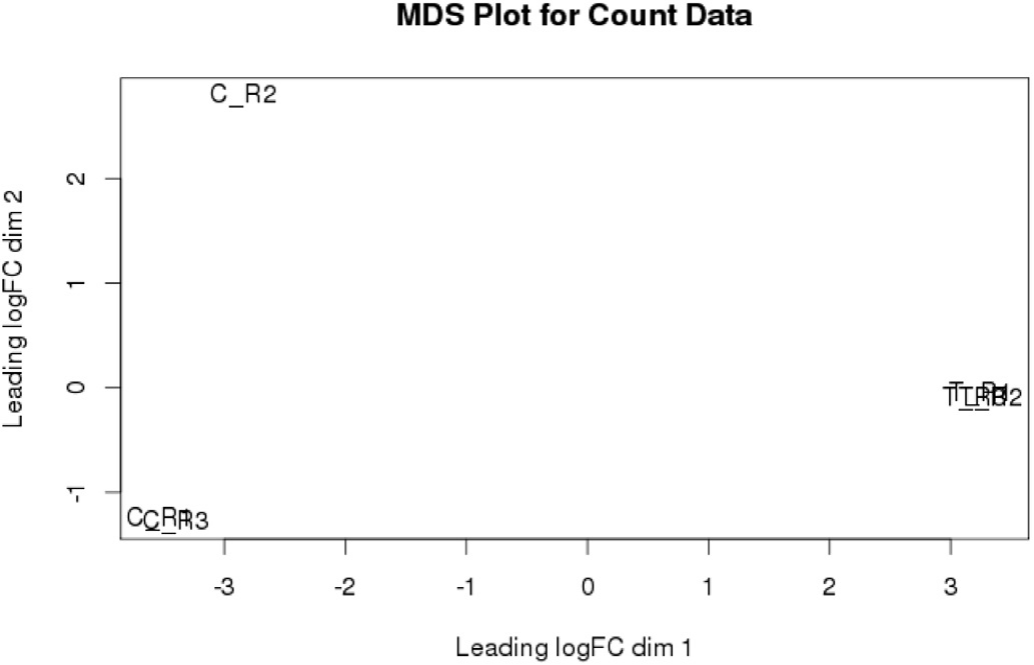
Plots of sample relations generated using a count-specific distance measure edgeR's function *plotMDS* which produces a multidimensional scaling plot showing the relationship between all pairs of condition specific samples (Control [C_R1, C_R2, C_R3] vs Treated [T_R1, T_R2, T_R3]).

Bioconductor package based tool edgeR (v3.3) (https://bioconductor.org/packages/release/bioc/html/edgeR.html) used read count values clustered in EXCEL sheets to identify significantly up- and down- regulated genes (Supplementary Table 1). The tool edgeR calculates the expression of sample using mean-values and dispersion of expression around this mean value. edgeR contains columns for log-fold change (logFC), counts per million (or mean by condition, CPM), likelihood ratio statistic (for GLM-based analysis), as well as raw and adjusted *P* values facilitating multiple testing and extracting feature-level information [31]. Total 61800 genes were obtained as expressed genes, among which using Poisson distribution 18498 genes showed DE. Both, the Tagwise and Common dispersion method showed 8767 DEGs, respectively. Among the total genes 3475 were found to be down-regulated; 4114 were found to be up-regulated and 10909 did not show any regulation, exhibiting significant alterations in expression by applying Fisher's exact test on a negative binomial (NB) distribution (Supplementary Table 2). Genes with positive and negative FC were considered significantly to be up- and down-regulated, respectively. The subsequent analysis steps were same for both the replicates. The relationships between samples using a multidimensional scaling (MDS) plot, was inspected using the function plotMDS and the generated graph is shown in Fig. 5.

**Fig. 6:**
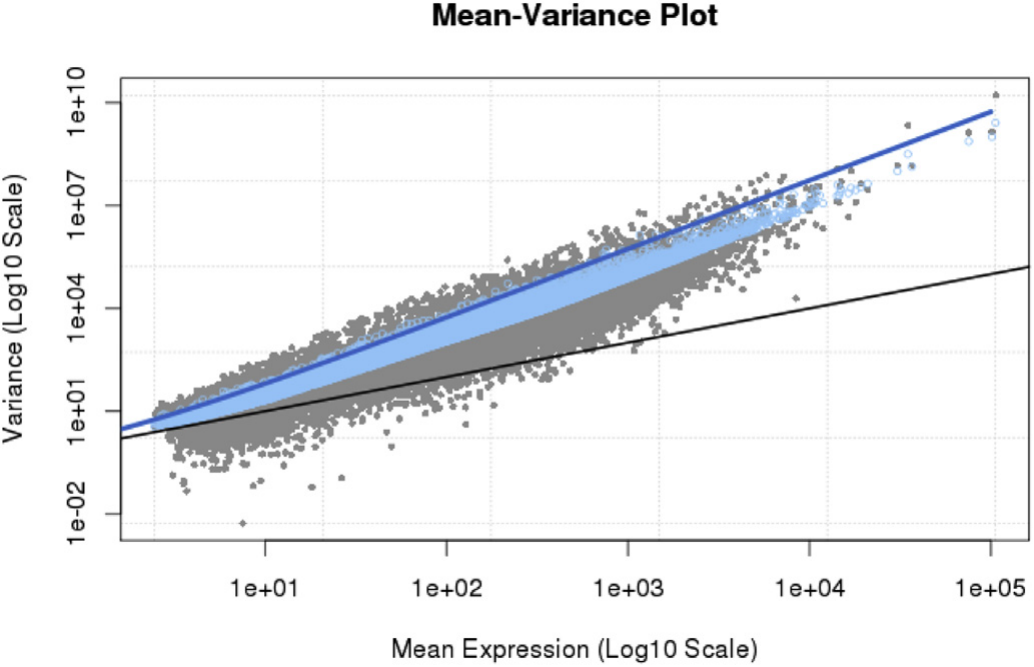
Plots of mean-variance relationship and dispersion was plotted using edgeR's plotMeanVar function to explore the mean-variance relationship. Each dot represents the estimated mean and variance for each gene, with binned variances as well as the trended common dispersion overlaid (Control vs Treated).

A visual representation of the mean-variance relationship using the plotMeanVar was generated as given in Fig. 6.

**Fig. 7:**
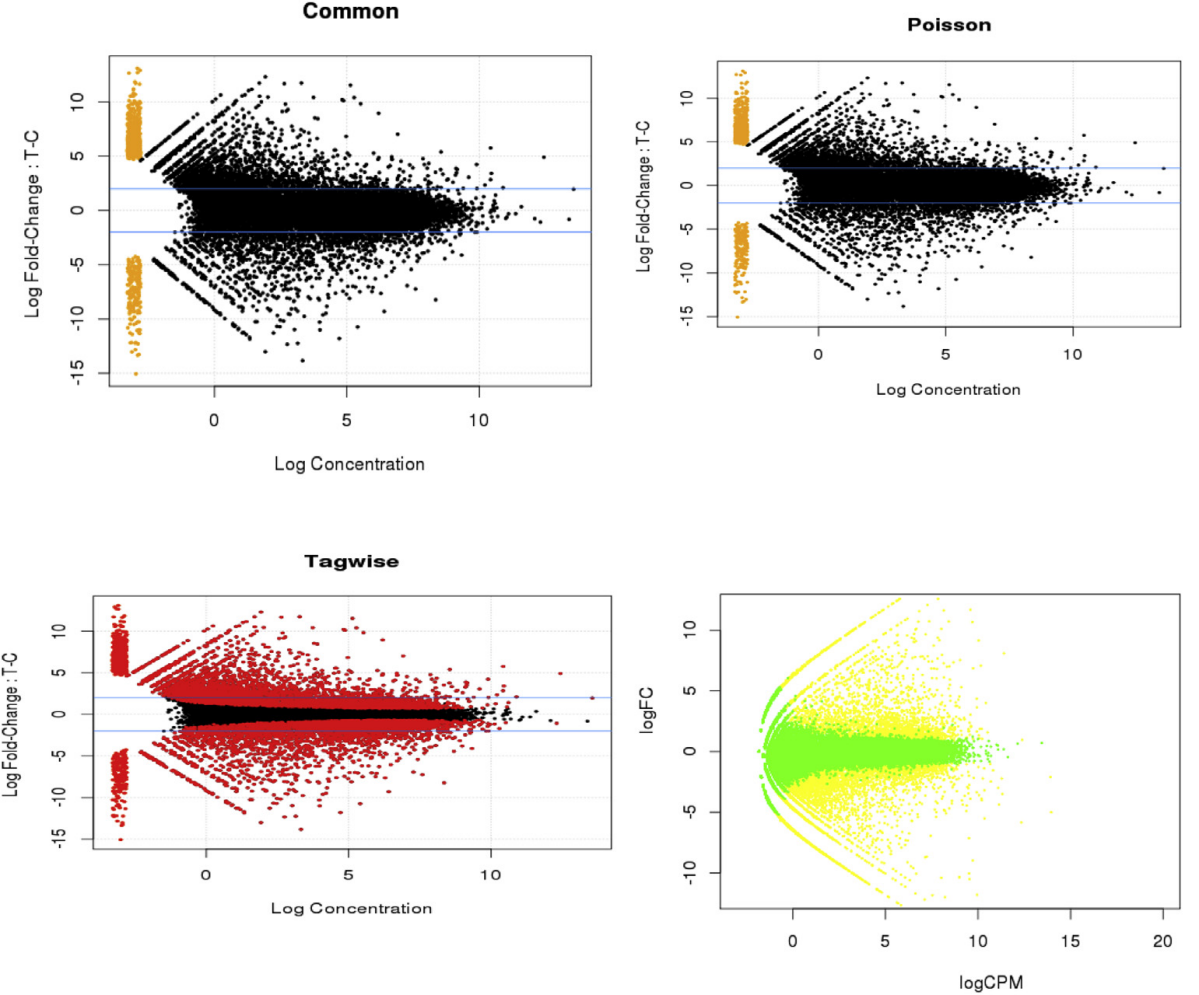
Control versus Treated plots for RNA-seq data for two conditions: cancer and normal. edgeR's plotSmear function plots the log-fold change (i.e., the log ratio of normalized expression levels between two experimental conditions) against the log concentration for (a) Common Dispersion, (b) Tagwise Dispersion and (c) Poisson Dispersion (d) edgeR's plotMA function plots the log-fold change (i.e., the log ratio of normalized expression levels between two experimental conditions) against the log counts per million (CPM).

The graph of differential expression results, such as logFC versus log-average expression value (calculated as a measure of read count) consisting of the genes selected as differentially expressed (with a 5% false discovery rate) were plotted using plotMA function of edgeR, as shown in Fig. 7(a, b, c, d). edgeR's plotSmear function was used to plot ‘Control versus Treated plots’ based on the log-fold change value (i.e., the log ratio of normalized expression levels between two experimental conditions) against the log concentration for (a) Common Dispersion, (b) Tagwise Dispersion and (c) Poisson Dispersion (d) edgeR's plotMA function plots the log-fold change (i.e., the log ratio of normalized expression levels between two experimental conditions) against the log counts per million (CPM).

## 3 Results

### 3.1 Sequence analysis and non-coding family classification

**Fig. 8:**
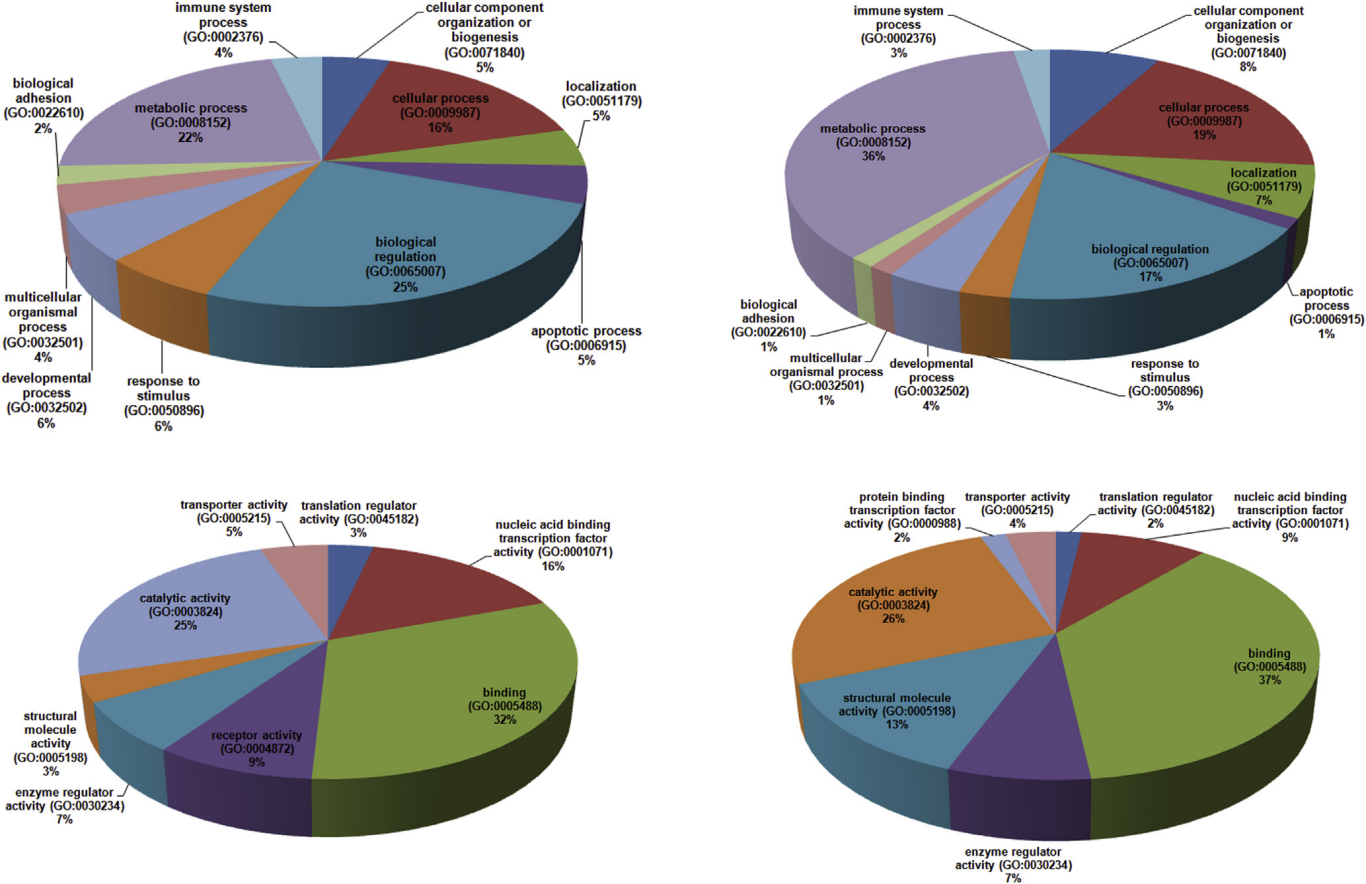
(a, b, c, d): Pie chart of the broad biological and molecular function associated with top 100 differentially expressed breast cancer genes/transcripts. Characterization of transcripts according to (a) biological functions for down-regulated transcripts, (b) molecular functions for down-regulated transcripts, (c) biological functions for up-regulated transcripts, and (d) molecular functions for up-regulated transcripts using PANTHER.

Functional annotation and characterization of DE lncRNAs obtained from RNA-seq was done to check their involvement in different biological and molecular functioning, using BiomaRt (an R package) (https://bioconductor.org/packages/release/bioc/html/biomaRt.html). Gene ontology (GO) classification was done for segregating significant genes. Further functional classification of the annotated unigenes in molecular function and biological process category was done using PANTHER classification system (http://www.pantherdb.org/) [32]. A list with associated ENSEMBL transcript ids containing functional classification of the genes, statistical over representation of the data and statistical enrichment test was generated. Deemed to be differentially expressed, the corresponding statistics can be used in downstream interpretive analyses to confirm or generate further hypotheses. Analysis of biological processes showed that several up-regulated genes were associated with metabolic process, biological regulation, and cellular process, whereas down-regulated genes were associated with metabolic process, cellular process and biological regulation, respectively. GO enrichment analysis for the DEGs showed that the up-regulated genes were mostly involved in molecular functions such as DNA binding, catalytic activity, and nucleic acid binding TF activity, and the down-regulated genes were implicated in DNA binding, catalytic activity and structural molecule activity, respectively (Fig. 8). Similar results were also observed using GOrilla pathway analysis (http://cbl-gorilla.cs.technion.ac.il/).

Functional and pathway assignments of the DEGs using Panther and GOrilla classification revealed numerous hormonal, physiological, and developmental changes in condition specific dataset. Genes related to DNA binding, catalytic activity, metabolism, regulation, cellular process, structural molecular activity and transcription were shown to be most regulated in tumorous conditions [33]. The present data suggested involvement of ncRNAs in establishing the metabolic equilibrium during tumorous condition to enable the role of lncRNAs in regulating mRNA as well as other ncRNAs.

### 3.2 Bioinformatics analysis of differentially expressed lncRNAs

Statistical over representation of the top 100 up-regulated and 100 down-regulated transcripts showed the name of annotation data category, number of genes uploaded list that mapped to the particular annotation category and the genes that did not map to the particular annotation data category. The top 100's for both the regulated categories were selected on the basis of logFC value (for up-regulated logFC was >9.24 and for down-regulated logFC was >−0.89) and could be considered statistically significant. The threshold value was based on *P* value, determined by binomial statistics. The probability of finding genes/transcripts in this category occurs randomly (by chance), as determined by the reference list provided. A small *P* value indicated that the number obtained is significantly rich and potentially of use. Only those lncRNAs with a *P* value of <0.05 was used as default parameter, were screened out for further analysis. Among top 100, total 57 up-regulated and 54 down-regulated transcripts were found to unmap the existing GO database with enriched coding genes (Supplementary Table 3). Some specific non-coding transcripts were searched against (a) noncoding RNA database (NONCODE) (http://www.noncode.org) and (b) LNCipedia database (http://www.lncipedia.org/*)* for identification and classification of these ncRNAs. Use of two different databases removed the chance of false annotation of non-coding genes.

It was found that unmapped/unannotated transcripts fall into the category of lncRNAs, miRNAs, snRNAs, snoRNAs and miscRNAs. This research was more focussed towards the identification of DE lncRNAs, therefore to strengthen our analysis the corresponding NONCODE ids were searched against LNCipedia database. Overall, 91% of up-regulated genes and 35% of down-regulated genes were classified as lncRNAs in top 100 differentially regulated dataset. Database search of lncRNAs emphasized that most of these DE lncRNAs were novel and lacked experimental and literature evidences to be reported in breast cancer. The information like location, strand, transcript size, sources, alternative transcript names, RNA-sequence, structure, protein coding potential, locus conservation, secondary structure conformation and targeting miRNAs were extracted from the database [19], [20]. About 19% (11 in numbers) of the up-regulated lncRNAs were found to have human locus conservation with mouse and 3% (2 in numbers) showed locus conservation with mouse and zebrafish, both.

**Fig. 9:**
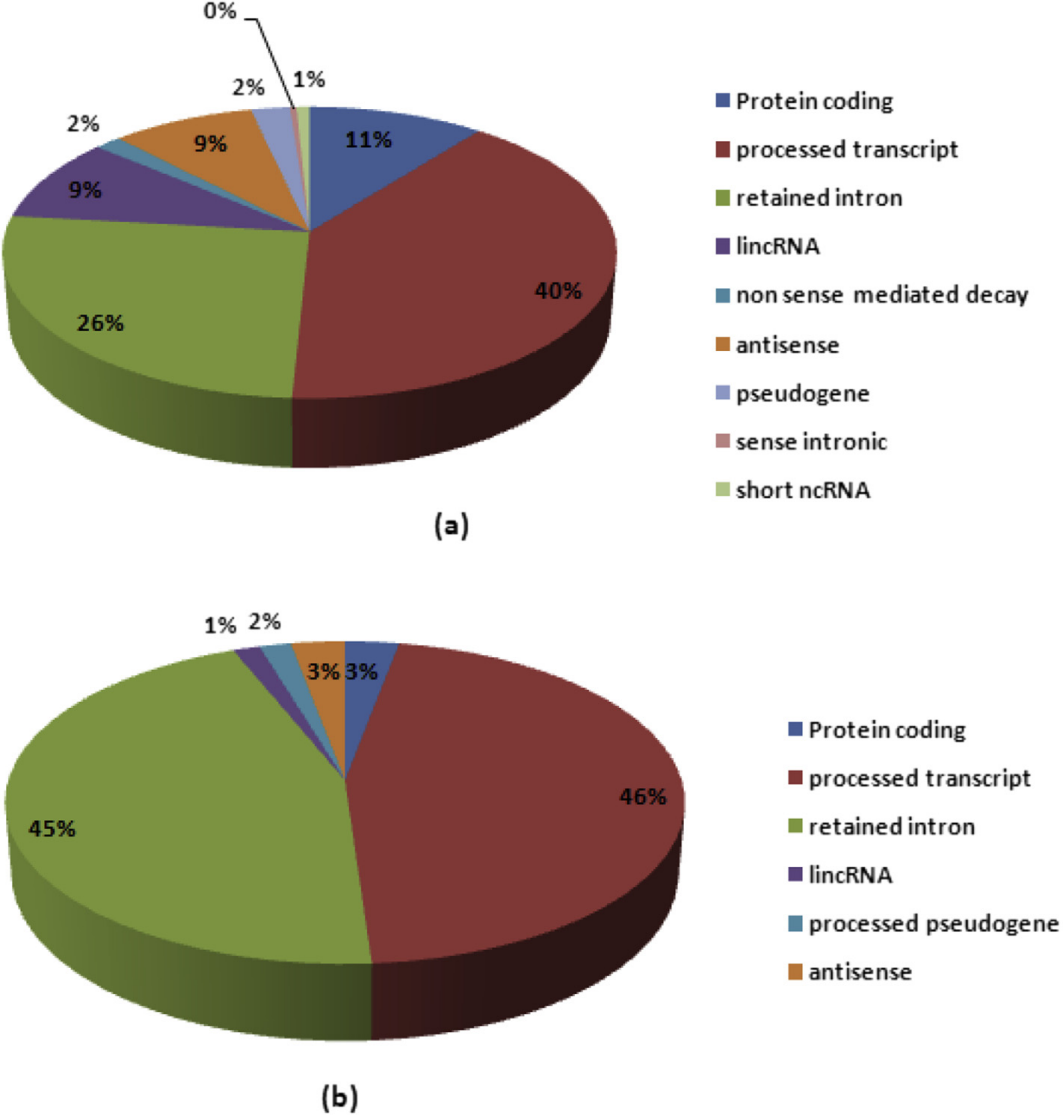
Characterization of the Transcriptome assembly. (**a**) Pie chart of the composition and quantities of Protein coding genes, processed transcript, retained intron, lincRNA, non sense mediated decay, antisense, pseudogene, sense intronic, and short ncRNAs in the up-regulated assembly. (**b**) Pie chart of the composition and quantities of Protein coding genes, processed transcript, retained intron, lincRNA, antisense, and processed pseudogene in the down-regulated assembly.

Remarkably, over 11% of lncRNAs were categorized as large intergenic non-coding RNAs (lincRNAs), pseudogene, unitary pseudogene, transcribed unprocessed pseudogene, unprocessed pseudogene, processed pseudogene, antisense and sense lncRNAs (Fig. 9a) in an up-regulated condition. In down-regulated conditions 6% of lncRNAs were classified as lincRNAs, processed pseudogene and antisense (Fig. 9b).

## 4 Discussions

The study aimed at identifying novel lncRNAs that are believed to be involved in breast cancer and governs the expression change of a cell. From the RNA-seq workflow, it was observed that the total number of DEGs in breast cancer was 18498 out of which 4114 were observed to be up-regulated and 3475 were down-regulated. And finally we could conclude that large number of lncRNAs showed DE patterns in condition specific dataset breast cells (such as control versus treated).

On comparing our result, with existing experimentally validated datasets obtained from various non-coding databases such as NONCODE and LNCipedia, we found that three lncRNAs, lnc-MTAP (*CDKN2B-AS1*), lnc-PCP4 (*DSCAM-S1*), lnc-FAM (*H19)* which was previously reported to be up-regulated in Breast cancer, were also up-regulated in our study. Other 51 novel lncRNAs which are not yet reported in breast cancer can help us to associate their aberrant expression pattern with the disease (the ENSEMBL Gene Id of the related lncRNAs has been provided in Supplementary Table 4). DE lncRNA, *H19* is also said to be involved in various other cancer types such as bladder cancer, cervical cancer, colon cancer, gastric cancer, kidney cancer, liver cancer, lung cancer and ovarian cancers (Reported in LncRNADisease database). Li H et al. reported in his paper that the over-expression of lncRNA *H19* increases the carcinogenesis and metastasis of gastric cancer [34]. Metastasis is one of the very well known hallmarks of cancer [35]; therefore detail analysis with validated result can help us to find the role of other novel lncRNAs discovered in our studies and associate them with other hallmarks of cancer like angiogenesis, apoptosis, etc.

*XIST,* a class of lncRNA is associated to both sex and non-sex related cancers. XIST's relationship with the breast cancer gene BRCA1 (which is a well-known tumour suppressor gene, TSG) has been widely studied. It has been found that BRCA1-defficient breast cancer cell lines have increased XIST expression suggesting it to be used as a marker to study tumour development [36]. These individual examples have been functionally studied, and many more important questions are yet to be addressed. The expression of H19 in healthy tissues as well as breast adenocarcinomas tissues can further be associated with breast cancer gene and the enhancement in tumorigenic properties of breast cancer cells can be studied.

Further detail research showed that *H19* and mir-675 functions in a similar manner [37]. Therefore, in future we can dedicate a section of our research in finding non-coding RNAs interaction with other non-coding RNAs. Regulation of one ncRNA by another ncRNA is a very challenging and demanding field of research for scientific community. The target pairs can thus be hypothesized to play role in breast cancer and the result can further be experimentally validated. The conventional interaction observed mostly is lncRNA targeting miRNA, however the interaction can also be in the reverse way. Also some other ncRNA can be involved in the regulation process. A successful research on this topic may help in unfolding many hidden ways of malignancy cause in the case of cancer.

The current research majorly focuses only on short ncRNAs, whereas the underestimated long ncRNAs have a great potential to be considered as important molecules in physiological as well as pathological settings. Therefore, the next step should be genome-scale identification of lncRNAs differentially expressed in wide variety of human cancers. We have discussed many lncRNAs with DE patterns that could be diagnostic, prognostic, or predictive value for various types of cancer. lncRNA offers a number of advantages as diagnostic and prognostic markers. The great wealth of newly discovered transcripts makes it highly likely that many other lncRNA markers remain to be discovered. *DE analysis study* has been successful in pinpointing new cancer-associated lncRNAs by following the approach of unbiased modality for gene discovery. By uncovering this expansive landscape of tissue- and cancer-associated lncRNAs, we provide the scientific community with a powerful starting point to begin investigating their biological relevance. The vast amount of cancer genome data becoming rapidly available can only be fully exploited if also the non-coding content of the human cancer genome is studied in great deal- after all it constitutes the large majority of the genomic information. The more we learn about lncRNA expression patterns in different types of cancer – as well as in healthy cells – the higher the chances for an improved diagnosis and better prognosis will be.
